# Spt6 levels are modulated by PAAF1 and proteasome to regulate the HIV-1 LTR

**DOI:** 10.1186/1742-4690-9-13

**Published:** 2012-02-08

**Authors:** Mirai Nakamura, Poornima Basavarajaiah, Emilie Rousset, Cyprien Beraud, Daniel Latreille, Imène-Sarah Henaoui, Irina Lassot, Bernard Mari, Rosemary Kiernan

**Affiliations:** 1 Laboratoire de Régulation des Gènes, Institut de Génétique Humaine, CNRS UPR1142, Montpellier, France; 2 Institut de Pharmacologie Moléculaire et Cellulaire, UMR6097 CNRS/UNSA, Sophia Antipolis, Nice, France; 3 University of Nice, Sophia Antipolis, Nice, France; 4 Current address: IGMM, Montpellier, France

**Keywords:** LTR, transcription, Tat, Spt6, PAAF1, proteasome

## Abstract

**Background:**

Tat-mediated activation of the HIV-1 promoter depends upon a proteasome-associated factor, PAAF1, which dissociates 26S proteasome to produce 19S RP that is essential for transcriptional elongation. The effect of PAAF1 on proteasome activity could also potentially shield certain factors from proteolysis, which may be implicated in the transcriptional co-activator activity of PAAF1 towards the LTR.

**Results:**

Here, we show that Spt6 is targeted by proteasome in the absence of PAAF1. PAAF1 interacts with the N-terminus of Spt6, suggesting that PAAF1 protects Spt6 from proteolysis. Depletion of either PAAF1 or Spt6 reduced histone occupancy at the HIV-1 promoter, and induced the synthesis of aberrant transcripts. Ectopic Spt6 expression or treatment with proteasome inhibitor partially rescued the transcription defect associated with loss of PAAF1. Transcriptional profiling followed by ChIP identified a subset of cellular genes that are regulated in a similar fashion to HIV-1 by Spt6 and/or PAAF1, including many that are involved in cancer, such as BRCA1 and BARD1.

**Conclusion:**

These results show that intracellular levels of Spt6 are fine-tuned by PAAF1 and proteasome, which is required for HIV-1 transcription and extends to cellular genes implicated in cancer.

## Background

Spt6 is a highly conserved transcription factor that plays a number of distinct roles during transcription. It interacts directly with histones, particularly H3, and possesses nucleosome assembly activity *in vitro *[1]. Together with FACT, a H2A/H2B chaperone, Spt6 restores chromatin structure in the wake of elongating RNAPII [2,3]. Loss of Spt6 disrupts normal chromatin structure and leads to the initiation of cryptic transcripts from within the coding region [3]. At inducible genes, Spt6 is required to restore transcriptional repression by promoting nucleosome reassembly over the promoter region [4,5]. Spt6 is a transcription elongation factor that is associated with the body of genes during transcription [6,7] and enhances the elongation rate of RNAPII, even on naked DNA [8,9]. It contains tandem SH2 domains that interact with phosphorylated Ser2 and Ser5 of the carboxy-terminal domain (CTD) of RNAPII [10,11]. Spt6 is also implicated in mRNA processing through interactions with the nuclear exosome subunit, Rrp6, and Iws1 that recruits RNA processing/export factors Ref1/Aly [11,12], and prevents premature 3' processing at cryptic, upstream polyadenylation sites [6]. Spt6 controls basal and Tat-mediated transcription from the HIV-1 promoter [13,14] and controls HIV-1 latency [15]. Thus, Spt6 is required for transcription through chromatin by ensuring proper nucleosome reassembly during elongation and linking transcription to mRNA processing and quality control.

26S proteasome, the major pathway of degradation for ubiquitinated proteins in cells, consists of two large subcomplexes, 19S regulatory particle (19S RP) and 20S core particle (20S CP) [16]. 19S RP recognizes polyubiquitinated substrates that are subsequently degraded by the 20S catalytic particle (20S CP) in an energy-dependent manner. Previous studies have highlighted a role for proteasome in controlling transcription [17]. Transcription-coupled proteolytic destruction of activators facilitates gene activation at certain promoters [18]. Other studies, by contrast, suggest that 19S RP positively affects transcription through mechanisms that are independent of proteolysis but may require the ATPase-dependent chaperonin activity of 19S RP [19-21]. Indeed, proteolytic activity is excluded from regions that are highly transcribed, but can become associated with such regions under conditions that lead to transcriptional stalling [22]. Alternatively, the degradation of transcription factors can be more specifically controlled in several ways. Most simply, the ubiquitinated lysine residue can also be an acceptor residue for acetylation. Thus, factor degradation is controlled via cycles of acetylation/deacetylation, as shown for Foxo3 and RelA [23,24]. In other cases, interaction with another factor can control the rate of substrate degradation. For example, interaction with FANCJ prevents proteolytic degradation of Blm [25]. Similarly, HSP70 protects ATF5 from rapid degradation by proteosome in glioma cells [26]. Thus, the proteolytic degradation of certain factors can be specifically and reversibly controlled, which has important implications for a number of cellular processes, including transcription.

We have previously demonstrated that transcription from the HIV-1 promoter is controlled by proteasome [27]. In the absence of the viral transactivator, Tat, 26S proteasome is associated with the promoter and represses basal transcription. In the presence of Tat, however, 19S RP is recruited to the HIV-1 promoter where it facilitates an early step in transcriptional elongation in a non-proteolytic manner. This switch is dependent on a proteasomal ATPase-associated factor, PAAF1. Originally identified through its binding to a 19S RP subunit, PAAF1 was shown to regulate proteasome assembly and activity [27,28]. PAAF1 and its yeast homologue, Rpn14, were subsequently characterized as 19S RP chaperones [29,30]. Since PAAF1/Rpn14 associates with the fully assembled base of 19S ATPases, but dissociates prior to association of 20S CP, it is not detected in association with 26S proteasome [28,31]. PAAF1 is physically associated with HIV-1 chromatin and regulates 26S proteasome dynamics to produce 19S RP that is essential for transcriptional elongation in the presence of Tat [27,28]. Since both 19S RP and 20S CP are present on the HIV-1 promoter in the absence of PAAF1, it seems likely that promoter-associated proteasome activity is enhanced under these conditions [27]. This raises the possibility that PAAF1 might shield certain factors from proteasomal degradation, which could have consequences for HIV-1 transcription. Here, we show that PAAF1 specifically protects Spt6 from proteasomal degradation, which is crucial for nucleosome assembly during transcription at the HIV-1 promoter. Since regulation of the LTR is often a paradigm for cellular genes, we wondered if this mechanism operating at the LTR extends to cellular genes. Transcriptional profiling in either PAAF1 or Spt6 knockdown cells followed by ChIP analysis at selected genes revealed an important role for Spt6 and PAAF1 in controlling the expression of a subset of genes involved in cancer.

## Results

### Spt6 level is modulated by PAAF1 in proteasome dependent-manner

**Figure 1:**
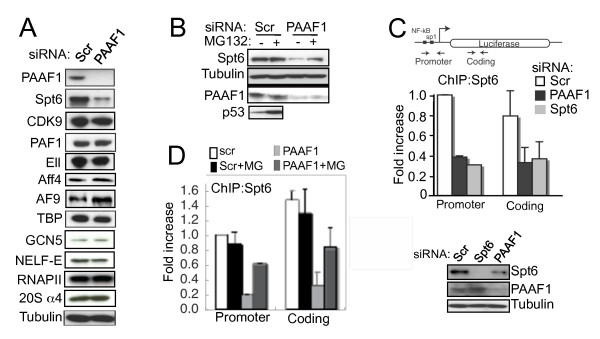
**Spt6 levels are controlled by PAAF1 in a proteasome-dependent manner**. (A) HeLa cells transfected with siRNA against PAAF1 or a control (Scr) were analyzed by IB using the indicated antibodies. (B) Extracts from HeLa transfected with the indicated siRNAs and treated with MG132 where indicated were analyzed by IB using the indicated antibodies. (C) Spt6 association with HIV-1 chromatin is diminished by ablation of PAAF1. HeLa-LTR-luc transfected with the indicated siRNAs were analyzed by IB using the indicated antibodies, and ChIP using anti-Spt6. Sequences within the HIV promoter and *luciferase *region were amplified by Q-PCR. The value for the Scr sample in the promoter region was set to 1. Locations of primers used for PCR amplification are indicated above the graph. (D) Cells in *B *were analyzed by ChIP as described in *C *using anti-Spt6. Graphs show mean +/- SD (n = 3).

PAAF1 is a modulator of proteasome [27,28] and 19S RP chaperone [29,29-31]. We have previously shown that PAAF1 enhances HIV-1 transcription through its ability to generate free 19S RP that facilitates transcriptional elongation [27]. To address whether the 19S chaperone function of PAAF1 might play an additional role in transcription by protecting certain factors from proteasomal degradation, we sought to identify factor(s) important for HIV-1 transcription whose stability is dependent on PAAF1. Following RNA interference (RNAi) against PAAF1 or a non-specific control (scr), a number of transcription factors were analyzed by IB to identify those diminished by loss of PAAF1. Among those analyzed, only Spt6 was significantly reduced in PAAF1 RNAi cells. Subunits of RNAPII, P-TEFb, PAF1 and NELF complexes, among others, were not affected (Figure 1A and Additional file 1, Figure S1A). In addition, PAAF1 did not affect the level of several known proteasome substrates (Additional file 1, Figure S1B) suggesting that the effect on Spt6 is not due to global deregulation of proteasome. While Spt6 protein was diminished in PAAF1 RNAi cells, no effect was observed on Spt6 mRNA as measured by RT-Q-PCR (Additional file 2, Figure S2), indicating that PAAF1-mediated modulation of Spt6 occurs post-transcriptionally. Next, to determine whether Spt6 is degraded by proteasome, we analyzed Spt6 levels in cells treated for 8 h with or without proteasome inhibitor, MG132, in the presence or absence of PAAF1. Accumulation of p53 in MG132-treated cells shows that proteasome was efficiently inhibited (Figure 1B). Diminution of Spt6 in PAAF1 RNAi cells was partially restored by treatment with MG132 (Figure 1B). These data suggest that PAAF1 protects Spt6 from proteasomal degradation, and modulates the intracellular level of Spt6 protein.

We then asked whether diminution of Spt6 following PAAF1 depletion might affect Spt6 recruitment to chromatin. Control or PAAF1 RNAi cells containing a stably integrated LTR linked to a Luciferase reporter (HeLa-LTR-luc) were analyzed by chromatin immunoprecipitation (ChIP) to detect Spt6 association with the HIV-1 promoter and *luciferase *region. Spt6 association was reduced to almost the same extent by PAAF1 RNAi as by Spt6 RNAi (Figure 1C). In contrast, Spt6 recruitment to GAPDH was not affected indicating that the effect might be restricted to specific genes (Additional file 3, Figure S3). Since the Spt6 level in cell extract was partially recovered by MG132, we asked if Spt6 recruitment to the LTR was also rescued. MG132 treatment significantly enhanced Spt6 association with HIV-1 chromatin in PAAF1 RNAi cells but had no effect in control cells (Figure 1D). These data suggest that PAAF1 stabilizes Spt6 to levels that are sufficient for its association with HIV-1 chromatin.

### PAAF1 physically interacts with Spt6

**Figure 2:**
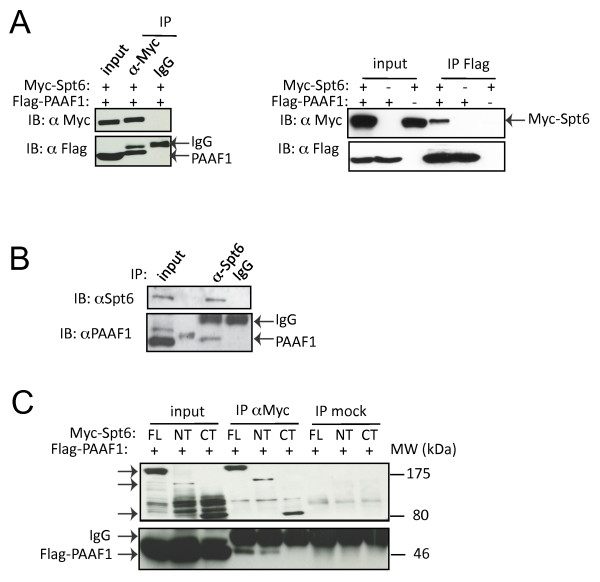
**PAAF1 interacts with the N-terminus of Spt6**. (A) Extracts from 293T transfected with PAAF1-Flag alone or together with Myc-Spt6 were analyzed by IP-IB using the indicated antibodies. (B) 293T extract was immunoprecipitated using anti-Spt6 followed by IB using anti-PAAF1. (C) Extracts from 293T transfected with PAAF1-Flag and the indicated Spt6-Myc constructs were immunoprecipitated using anti-Myc or IgG followed by IB with anti-Flag.

**Figure 3:**
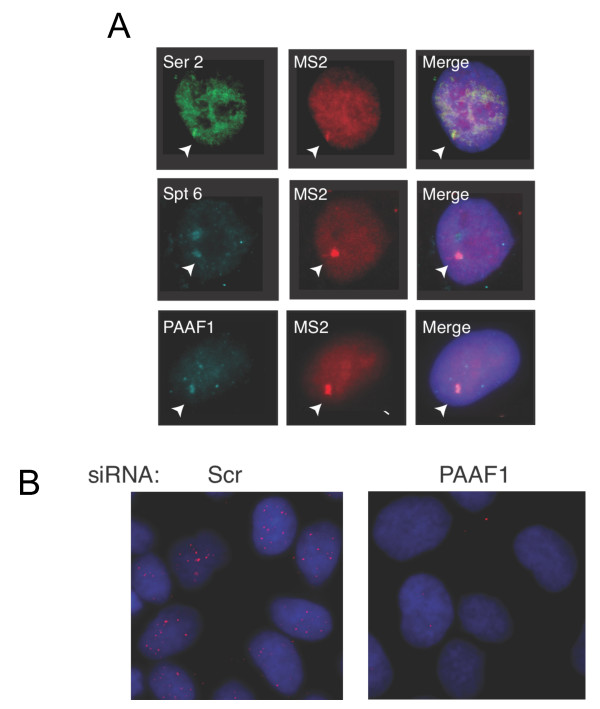
**Spt6, PAAF1 and RNAPII localize at sites of HIV-1 transcription**. (A) U2OS-LTR-MS2 cells transfected with pMS2-cherry and pTat-Flag were fixed, and analyzed by immunofluorescence using the indicated antibodies. Sites of co-localization with MS2-labelled HIV-1 transcription sites are indicated by arrowheads. (B) Anti-PAAF1 antibody specifically recognizes endogenous PAAF1. U2OS-LTR-MS2 cells were transfected with control (scr) or PAAF1-specific siRNA, and fixed 48 hr later. Cells were pre-treated with 0.05% Triton/PBS, fixed and stained with anti-PAAF1 antibody (red) for immunofluorescence analysis.

Since PAAF1 modulated Spt6 levels without affecting other factors, we tested whether these two proteins interact. Extracts from 293T cells expressing Flag-PAAF1 alone or together with Myc-Spt6 were used for co-immunoprecipitation analysis. Flag-PAAF1 was immunoprecipitated by anti-Myc antibody but not control IgG (Figure 2A, left panel). Similarly, Myc-Spt6 was immunoprecipitated by anti-Flag antibody (Figure 2A, right panel). Furthermore, an interaction between endogenous Spt6 and PAAF1 could also be detected in 293T extract (Figure 2B). To identify the region of Spt6 that binds to PAAF1, full-length and mutant Myc-Spt6 were transiently expressed in 293T cells and analyzed for associated PAAF1 by IB (Figure 2C). PAAF1 bound to the N-terminal (1-916) fragment of Spt6, but did not recognize its C-terminal fragment (1162-1726) that interacts with RNAPII [11]. Thus, PAAF1 specifically interacts with the N-terminal region of Spt6. We then asked whether these factors localize to sites of HIV-1 transcription. Transcripts emanating from the HIV-1 LTR were visualized using U2OS cells in which an LTR, which is linked to a reporter containing binding sites for MS2 protein, is stably integrated (U2OS-LTR-MS2) [32]. Following activation of HIV-1 by Tat, HIV-1 transcription foci are marked by an accumulation of MS2-cherry bound to its cognate binding sites present in the HIV-1 transcript (Figure 3A); elongating RNAPII that is phosphorylated on Ser2 of the CTD localized with the site of HIV-1 transcription. Spt6 similarly localized with HIV-1 transcription foci. Staining of endogenous PAAF1 revealed a diffuse nucleoplasmic pattern as well as several nuclear foyers. A PAAF1-containing foyer localized with the HIV-1 transcription site in the majority of cells examined. The specificity of the anti-PAAF1 antibody was validated in PAAF1 knockdown cells (Figure 3B). Thus, PAAF1, Spt6 and elongating RNAPII localize to sites of HIV-1 transcription.

### PAAF1-mediated Stabilization of Spt6 Facilitates Nucleosome Reassembly during Transcription

**Figure 4:**
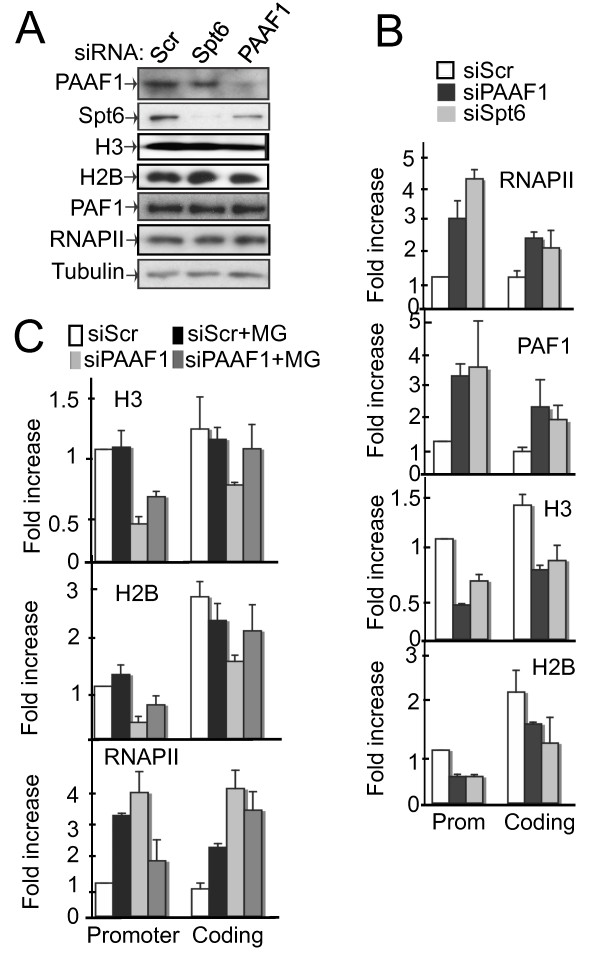
**Loss of PAAF1 or Spt6 leads to histone depletion and recruitment of RNAPII at HIV-1 sequences**. (A) HeLa-LTR-luc transfected with siRNA against Spt6, PAAF1 or a control were analyzed by IB using the indicated antibodies. (B) Cells in A were analyzed by ChIP using the indicated antibodies on the HIV promoter (prom) and *luciferase *region (coding) as indicated. The value for the Scr sample in the promoter region was set to 1. (C) HeLa-LTR-luc transfected with Scr or PAAF1 siRNA, followed by treatment with MG132 as indicated, were analyzed by IB and ChIP using the indicated antibodies. Sequences within the HIV-1 promoter and *luciferase *regions were amplified by Q-PCR. Values obtained for siScr samples in the promoter region were set to 1. Graphs show mean +/- SD (n = 3).

Spt6 is a histone chaperone that helps to restore chromatin structure during transcription [2,3]. Thus, control, Spt6 and PAAF1 knockdown cells were analyzed by ChIP for the association of histones H3 and H2B. Global levels of histones present in cell extract were not affected by the knock-downs (Figure 4A). However, both H3 and H2B were significantly diminished at the HIV-1 promoter (prom) and *luciferase *region by either PAAF1 or Spt6 RNAi (Figure 4B). In contrast, no effect on histone occupancy was observed at the *GAPDH *promoter region (Additional file 4, Figure S4). These results suggest that PAAF1 and Spt6 are required for histone reassembly at HIV-1 chromatin, but appear to be dispensable at other regions, such as *GAPDH*. The observed histone loss led us to analyze the recruitment of RNAPII to the HIV-1 promoter in PAAF1 and Spt6 knockdown cells. RNAPII association with the LTR and coding region was significantly enhanced in PAAF1 and Spt6 knockdown cells compared to controls (Figure 4B). Recruitment of PAF1, which associates with elongating RNAPII [33], mirrored that of RNAPII in PAAF1 and Spt6 knockdown cells (Figure 4B). Levels of RNAPII or PAF1 in cell extract were not significantly altered by knock down of either PAAF1 or Spt6 (Figure 4A). These data suggest that loss of PAAF1 destabilizes Spt6, which disrupts nucleosome reassembly at HIV-1 chromatin. Proteasome inhibition in PAAF1 knockdown cells partially recovered levels of Spt6 in cell extract (Figure 1B) and increased its association with HIV-1 chromatin (Figure 1D). The same samples were then used to address the role of PAAF1 and proteasome on nucleosome assembly through the stabilization of Spt6. Occupancy of H3 and H2B at HIV-1 chromatin was partially restored in PAAF1 knockdown cells following treatment with MG132 (Figure 4C) but was not affected in control cells. We have previously shown that MG132 increases transcriptional output from the HIV-1 promoter [27]. Consistent with this, RNAPII association with HIV-1 chromatin increased in control cells following MG132 treatment, in a PAAF1-independent manner (Figure 4C). While PAAF1 knockdown also enhanced RNAPII recruitment, as observed in Figure 4B, treatment of these cells with MG132 reset RNAPII levels closer to those in control cells (Figure 4C). Thus, inhibition of proteasome activity partially restored Spt6 levels and reversed the phenotype associated with loss of PAAF1. These data suggest that PAAF1-mediated modulation of proteasome activity stabilizes Spt6 and is required for nucleosome reassembly at the HIV-1 promoter.

### PAAF1-dependent stabilization of Spt6 is required to suppress aberrant HIV-1 transcription

**Figure 5:**
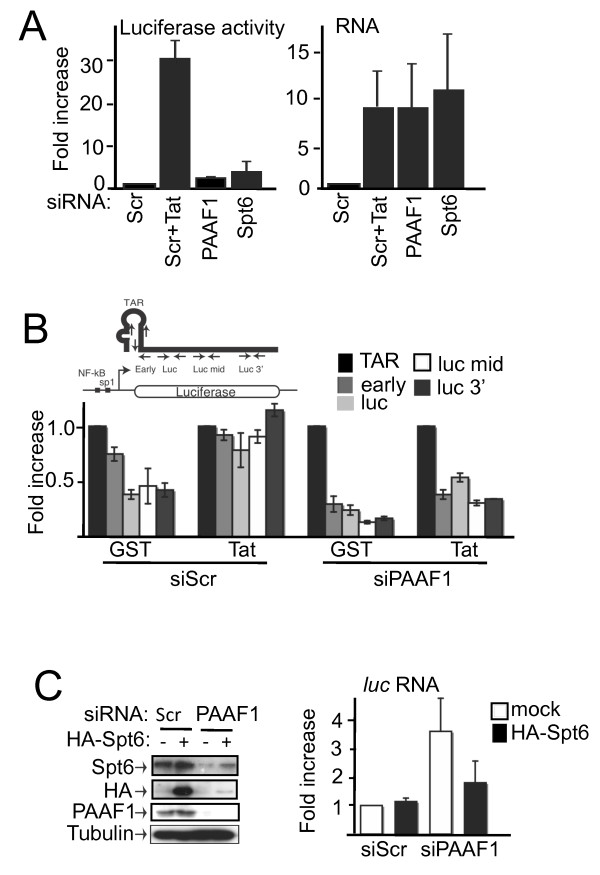
**Loss of Spt6 or PAAF1 induces aberrant HIV-1 transcript synthesis**. (A) Extracts of HeLa-LTR-luc cells transfected with siRNA against PAAF1, Spt6 or a control (Scr) and subsequently transduced with GST-Tat (Tat) as indicated were analyzed by IB using the indicated antibodies, Luciferase activity that was normalized to protein content, and RT-Q-PCR using Luc primers, which was normalized to the quantity of GAPDH mRNA in each sample. Values obtained for siScr samples were set to 1. (B) Reverse transcripts in A were analyzed by Q-PCR with primer pairs spanning the transcript. A schematic diagram of primer locations is shown above the graph. Transcript is shown as a black line containing the TAR RNA stem-loop at the 5' end. Values were normalized to the amount of GAPDH mRNA for each sample, and the value obtained for the TAR region was set to 1 for each condition. Graphs show mean +/- SD (n = 3). (C) Increase of HIV-1 transcripts following ablation of PAAF1 can be partially suppressed by exogenous Spt6 expression. HeLa-LTR-luc cells were transfected with Scr or PAAF1 siRNA and HA-Spt6 or mock expression vector, as indicated. Total RNA was isolated and analyzed by RT-Q-PCR using primers in the coding region. Graph shows mean +/- SD (n = 3).

Disruption of chromatin structure following loss of Spt6 function facilitates the initiation of cryptic transcripts from within the coding region [3]. Furthermore, loss of nucleosome reassembly in Spt6 mutant strains has been shown to increase *PHO *and *CHA1 *transcription in the absence of transcriptional activator [4,5]. To investigate the relevance of PAAF1-mediated protection of Spt6 in HIV-1 transcription, we analyzed HIV-1 transcript synthesis following PAAF1 or Spt6 knockdown. In control cells transduced with the HIV-1 transactivator, Tat, (Scr+Tat), *luciferase *mRNA and corresponding Luciferase activity was significantly increased (Figure 5A). PAAF1 knockdown cells showed a similar increase in transcript synthesis from the LTR, even in the absence of Tat. However, Luciferase activity was much reduced compared to Tat-treated samples. Like PAAF1 RNAi cells, loss of Spt6 increased transcript abundance similarly to Tat treatment, whereas Luciferase activity was relatively low (Figure 5A). A similar increase in transcripts in the absence of activator was also detected using U2OS-LTR-MS2 cells following loss of PAAF1 (Additional file 5, Figure S5). Neither PAAF1 nor Spt6 knockdown significantly affected GAPDH transcript abundance (data not shown).

The uncoupling of HIV-1 transcript synthesis and protein production in PAAF1 and Spt6 knockdown cells suggests that, although transcription is increased, the transcripts may be aberrant. RT-Q-PCR analysis was thus performed in the case of PAAF1 RNAi using primer pairs spanning the transcript (Figure 5B). Amounts of transcripts were normalized to the value obtained in the TAR region, a RNA hairpin located at the 5' end of initiated HIV-1 transcripts, in order to assess the efficiency of transcription elongation. In the absence of Tat (siScr + GST), transcripts were poorly elongated since more transcripts contained TAR than luc 3' sequences. As expected, transcripts were efficiently elongated in the presence of Tat since equivalent amounts of TAR and products up to the 3' end of *luciferase *could be detected. In PAAF1 knockdown cells, by contrast, transcripts were poorly elongated, in both the presence and absence of Tat (Figure 5B). Additionally, transcripts that were elongated up to the 3' end of *luciferase *were presumably incompetent for protein synthesis, which may be in part due to the function of Spt6 in mRNA 3' processing [6,7] export [11]. Thus, transcripts synthesized in the absence of PAAF1 are aberrant, likely due to a combination of defects in elongation and RNA processing/export. We next asked whether the transcriptional defect in PAAF1 knockdown cells could be suppressed by Spt6. Ectopic expression of Spt6 had no effect on HIV-1 transcription in control cells (Figure 5C). In contrast, expression of HA-Spt6 partially reversed the increase in transcription induced following loss of PAAF1. Taken together, these data show that Spt6 is required for repression of basal transcription, and loss of Spt6 following PAAF1 knockdown permits aberrant transcription that is highly inefficient for protein synthesis.

### Regulation of Cellular Genes by PAAF1 and/or Spt6

**Figure 6:**
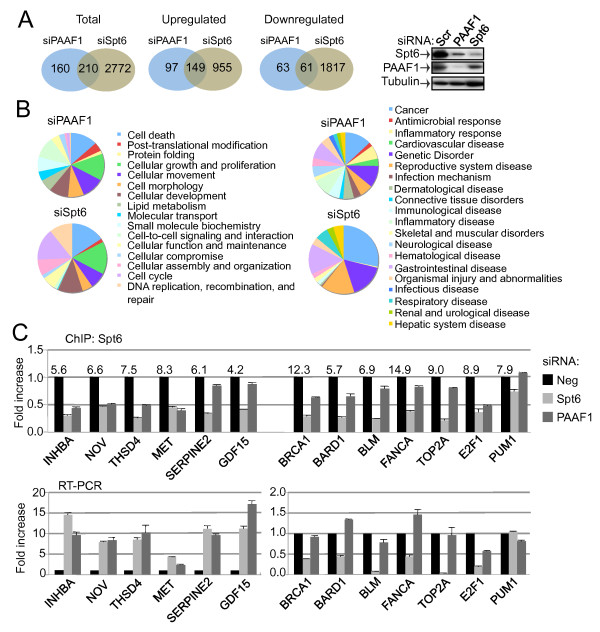
**Identification of cellular genes controlled by Spt6 and/or PAAF1**. (A) Total RNA extracted from HeLa cells transfected with control, Spt6 or PAAF1 siRNA was compared using pangenomic human microarrays. Shown are Venn diagrams representing the number of differentially expressed genes using a cut-off of 1 (log2). (B) Ingenuity pathway analysis of differentially expressed genes following Spt6 or PAAF1 RNAi. (C) Individual genes within the cancer pathway that were identified by transcriptional profiling were analyzed by ChIP using anti-Spt6, and RT-Q-PCR in HeLa cells transfected with control, PAAF1 or Spt6 siRNA. Graphs show mean +/- SEM (n = 3).

We next wished to determine whether PAAF1 and/or Spt6 regulate cellular genes in a similar fashion to the HIV-1 LTR. Thus, transcriptional profiling was performed using mRNA isolated from control, PAAF1 or Spt6 knock-down cells. When compared to the control sample, PAAF1 knockdown deregulated a specific subset of genes, of which more than 50% (210/370) were also deregulated by Spt6 knockdown (Figure 6A,Additional file 6, Figure S6A). This suggests that modulation of Spt6 protein level is probably a major function of PAAF1. Spt6 RNAi led to deregulation of a much larger subset of genes. Consistent with this, although the expression level of Spt6 was strongly diminished by siRNA against PAAF1, it is more severely reduced by Spt6-specific RNAi, as might be expected (Figure 6A). Gene ontology analysis revealed that knockdown of Spt6 or PAAF1 impacted most strongly on genes involved in cell growth and proliferation while Spt6 RNAi also affected genes involved in cell cycle and DNA replication, recombination and repair (Figure 6B). Since such genes are frequently deregulated in cancer, Ingenuity Pathway analysis showed that knockdown of PAAF1 or, more particularly, Spt6, impacted strongly on genes involved in cancer (Figure 6B). A group of genes was selected for further analysis. Spt6 association with selected genes was analyzed by ChIP in control, PAAF1- and Spt6 knockdown cells. Spt6 was enriched between 4 and 15-fold compared to a mock IP at all genes tested (Figure 6C). In Spt6 RNAi cells, its association was reduced between 2 and 4 fold at all genes except PUM1. In all cases, loss of Spt6 correlated with deregulation of transcription. However, the outcome was not the same at all genes examined. Transcript abundance was increased for INHBA, NOV, MET and others, while it decreased for genes including BRCA1, BARD1 and BLM (Figure 6C). Interestingly, PAAF1 knockdown reduced Spt6 association with some genes but not others. For example, Spt6 association with INHBA, NOV, THSD4, MET or E2F1 was diminished to almost the same extent in either PAAF1 or Spt6 knockdown cells (Figure 6C). Consistently, the effect on transcript abundance for each gene following PAAF1 or Spt6 knockdown was similar (Figure 6C). Exceptions to this were SERPINE2 and GDF15, which were enhanced by PAAF1 knockdown even though Spt6 association was unaffected. This group of genes was identified by transcriptional profiling as being regulated by both Spt6 and PAAF1. On the other hand, association of Spt6 with genes such as BRCA1, BARD1, BLM, FANCA and TOP2A was diminished by knockdown of Spt6 but not PAAF1. Consistently, transcript abundance was altered by RNAi against Spt6 but not PAAF1. These genes were identified by transcriptional profiling as being regulated only by Spt6. IB analysis of some of the factors analyzed by ChIP and RT-Q-PCR showed that the effects on transcript level correlated well with protein level in Spt6 knockdown cells (Additional file 6, Figure S6B). In PAAF1 knockdown cells, some effects on protein expression could be observed that appear to be post-transcriptional, for example for FANCA and TOP2A, in which transcript abundance was not reduced but protein level was nevertheless diminished. These effects may be due in part to the effect of PAAF1 on 26S proteasome activity that may alter the levels of factors that can be degraded by proteasome. Overall, this analysis demonstrates that Spt6 regulates the transcription of a number of genes that have been linked to cancer. However, loss of Spt6 produced different outcomes at different genes. Transcript levels of several oncogenes such as NOV and MET increased, while those corresponding to a number of factors involved in DNA repair, such as BRCA1, BARD1 and BLM, were diminished.

## Discussion

We have previously demonstrated that PAAF1, a modulator of proteasome activity, is required for HIV-1 transcription [27]. In this study, we show that PAAF1 specifically protects Spt6, a key factor in nucleosome reassembly, transcription elongation and RNA processing, from proteasomal degradation. PAAF1 interacts with Spt6 and both factors localize at the HIV-1 promoter. The level of Spt6 in cells, and also its association with HIV-1 chromatin are modulated by PAAF1 in a proteasome-dependent manner. Ablation of either PAAF1 or Spt6 led to loss of histones from HIV-1 chromatin, concomitant association of RNAPII and the induction of transcripts that were largely defective for protein synthesis.

As nucleosomes present a barrier to the passage of RNAPII, many factors, including histone chaperones, are required to coordinate their removal ahead of RNAPII, and their redeposition in the wake of elongating RNAPII [34]. The repositioning of nucleosomes can prevent cryptic transcription that may have deleterious effects. Loss of nucleosomes can deregulate transcription. For example, depletion of histone H4 increased transcription from a subset of genes in yeast [35], and reassembly of nucleosomes is required to repress transcription under non-inducing conditions [4]. Nucleosome reassembly requires the concerted action of chaperones, Spt6 and FACT, which interact with histones to restore chromatin structure in the wake of RNAPII. Interestingly, PAAF1 knockdown did not affect occupancy of the Spt16 subunit of FACT at HIV-1 chromatin suggesting that, while PAAF1 regulates Spt6, Spt16 is not subject to the same regulatory mechanism even though both chaperones frequently function at the same genes. Our findings indicate that modulation of Spt6 by PAAF1 and proteasome controls transcription at the HIV-1 promoter by facilitating the restoration of chromatin structure.

Transcriptional profiling following PAAF1 or Spt6 RNAi showed that, among genes that are deregulated by PAAF1 knockdown, more than 50% were also deregulated following Spt6 knockdown. Since the involvement of PAAF1 in transcription is not as widespread as Spt6, it suggests that additional factors may determine which of the Spt6-regulated genes are also regulated by PAAF1. Genes that are commonly deregulated may be those that are highly sensitive to reduced levels of Spt6 in cells. Gene ontology analysis revealed that many of the differentially expressed genes following either PAAF1 or Spt6 knockdown are implicated in cancer. Among the genes deregulated PAAF1 or Spt6 RNAi cells were several oncogenes, such as NOV and MET, whose expression was increased. In contrast, a subset of genes including tumor suppressors, such as BRCA1, BARD1 and BLM, were highly deregulated by loss of Spt6 but were only modestly affected or unaffected by loss of PAAF1, even though Spt6 level in these cells was significantly diminished. Since Spt6 is associated with all of the genes analyzed, the results suggest that certain genes, such as INHBA, NOV and MET may be highly sensitive to loss of Spt6, whereas others such as BLM, FANCA and TOP2A, may be more resistant to loss of Spt6 and can tolerate modest reductions in Spt6 expression levels. Thus, Spt6 appears to control the expression of a number of genes, including several that are involved in cancer.

## Conclusion

We show that the HIV-1 transcriptional coactivator, PAAF1, specifically protects Spt6, a key factor in nucleosome reassembly, transcription elongation and RNA processing, from proteasomal degradation, and is thus required for transcription from the HIV-1 LTR.

## Methods

### Cell culture, antibodies and plasmids

HeLa-LTR-luc cells that contain *luciferase *under the control of an integrated HIV-1 LTR were obtained from K.-T. Jeang (NIAID, NIH, USA) and propagated in Dulbecco's modified Eagle's medium (DMEM, Lonza) supplemented with 10% FBS and antibiotics. The cells were treated with MG132 (Sigma) for 8 h, where indicated. U2OS cells containing a stably integrated HIV-1 LTR linked a reporter containing binding sites for MS2 protein [32] were obtained from E. Bertrand (IGMM, Montpellier) and propagated in DMEM supplemented with 10% FBS and antibiotics. Antibody recognizing human PAAF1 was raised in rabbits against an immunogenic peptide (Abnova). Antibodies used were anti CDK9, p53, NOV, BARD1 and BLM (SCBT), RNAPII, H3, H2B, Spt6, PAF1 and TOP2A (Abcam), Spt6 for IP (Bethyl Laboratories), Tubulin and Flag M2 (Sigma). pcDNA3-Flag-Spt6-HA [9] and pMyc-Spt6 [11] were gifts from H. Handa and K.A. Jones, respectively. pTat-Flag has been described previously [27].

### RNAi and transfection experiments

HeLa LTR-Luc cells were transfected using Interferin (PolyPlus Transfection) with 5 nM double-stranded siRNAs following the manufacturer's instructions. At 48 hr after transfection, cells were treated with GST or GST-Tat as previously described [32]. Luciferase activity was measured 48 hr after transduction according to the manufacturer's protocol (Promega). Luciferase activity was normalized to protein concentration using Bradford (BioRad). Double stranded RNA oligonucleotides directed against target sequences in PAAF1 (AGC CUG UUC UCU GGA GGA A) [27], Spt6 (GAA GCC UCA UGU AGU GAC A), Cdk9 (CUA GGG CUC UUG UGU UUU U), and a control siRNA (Scrambled) [27] were purchased from Eurofins MWG Operon.

### Microarray Experiments

HeLa cells were transfected with 5 nM of a control siRNA (Ambion #1 Silencer select), or siRNAs targeting PAAF1 or Spt6 (modified by Silencer, Ambion). To prepare samples, total RNAs were extracted using Trizol (Invitrogen) and quantified by nanodrop spectrophotometry. RNA quality was evaluated using the Agilent Bioanalyzer 2100 and Lab-on-Chip Nano 6000 chip (ratio of the 28S/18S RNA ≥ 1.5). RNA samples from knockdown experiments were labeled with Cy3 dye using the low RNA input QuickAmp kit (Agilent) as recommended by the supplier. 825 ng of labeled cRNA probe were hybridized on a 8x60K high density SurePrint G3 gene expression human Agilent microarray. Two biological replicates were performed for each comparison. The experimental data have been deposited in the NCBI Gene Expression Omnibus (GEO) (http://www.ncbi.nlm.nih.gov/geo/) under series record GSE32033. Normalization was performed using the Limma package available from Bioconductor (http://www.bioconductor.org). Inter-slide normalization was performed using the quantile methods. Means of ratios from all comparisons were calculated and B test analysis was performed. Ontologies attached to each modulated gene were then used to classify them according to main biological themes using Ingenuity software (http://www.ingenuity.com/).

### RT-Q-PCR

Total RNA was extracted from HeLa LTR-luc cells using Trizol (Invitrogen) and reverse-transcribed using Superscript First-strand Synthesis System for RT-PCR (Invitrogen). RT products were amplified by quantitative PCR on a LightCycler LC480 (Roche) by using the oligonucleotides shown in Additional file 7, Table S1. TAR, early, luc and luc 3' and GAPDH primers have been described previously [27,32]. Q-PCR cycling conditions are available on request.

### Chromatin immunoprecipitation (ChIP)

HeLa-LTR-Luc cells were treated for 4 hr with DMEM containing 100 μM chloroquin (Sigma), and 2 mg/ml of GST or GST-Tat. ChIP analysis was performed as described previously [27], except using Dynabeads A or G (Invitrogen) for immunoprecipitation. Normal IgA or IgG (SCBT) was used as a negative control (mock sample). Quantification of immunoprecipitated material was performed by quantitative PCR using LightCycler LC480 (Roche) and normalized for input DNA. Sequences of oligonucleotide primers are shown in Table S1. LTR prom and coding primers have been described previously [27,32]. Primers were mixed with Quanti Tect Sybr Green **(**Qiagen), Biorad SYBR green (BioRad) or LightCycler LC480 SYBR Green I Master (Roche). Q-PCR cycling conditions are available on request.

Results were calculated as follows: the value obtained using the specific antibody, expressed as a percentage of input DNA, was divided by the value obtained for the mock IgA or IgG sample. The value obtained for the control sample, usually siScr, was set to 1. The test samples were expressed as a ratio of the control.

### MS2 Immunofluorescence

U2OS cells containing the HIV-LTR fused to 24 bacteriophage MS2-binding sites [36] were plated on coverslips and transfected with siRNA using Oligofectamine (Invitrogen). After 24 h, cells were transfected with vectors expressing Tat and MS2-GFP using Lipofectamine2000 (Invitrogen). For single MS2 staining, cells were washed once with PBS, and fixed with 4% paraformaldehyde (PFA, Sigma) for 20 min at room temperature, followed by DNA staining using Hoechst (Sigma). For immunofluorescence, cells were pre-permeabilized with 0.05% Triton-X-100 (Sigma) prior to fixation with 2 or 4% PFA for 5 min on ice. After a PBS wash, coverslips were incubated with blocking buffer (5% bovine serum albumin (BSA)/PBS) for 1 h at room temperature. Blocking buffer containing primary antibodies was overlaid onto a coverslip and incubated for 1 h at room temperature. After washing three times with PBS, cells were treated with secondary antibodies conjugated with Alexa488 or Cy5 (Invitrogen) in blocking buffer for 1 h at room temperature. After 3 PBS washes, samples were counterstained for DNA. Fluorescent images of fixed cells were imaged on a Leica DM 6000-1 microscope (Leica) using MetaMorph software (Molecular Devices).

## Competing interests

The authors declare that they have no competing interests.

## Authors' contributions

Contribution: MN, BM and RK designed the research; MN, PB, ER, CB, DL, ISH, IL and BM performed research and collected data, MN, BM and RK analyzed data and wrote the paper. All authors read and approved the final manuscript.

## Supplementary Material

Additional file 1 **Figure S1**. PAAF1 knock-down does not affect known proteasome substrates or transcription factors other than Spt6. HeLa cells were transfected with siRNA targeting a control (Scr) or PAAF1 (A), followed by treatment with proteasome inhibitor, MG132 (MG), for 8 h before harvesting, where indicated (B). Total cell extract was analyzed by IB using the indicated antibodies. Click here for file

Additional file 2 **Figure S2**. Spt6 levels are controlled by PAAF1 in a proteasome-dependent manner. Total RNA was isolated from cells in Figure 1A was analyzed by RT-Q-PCR using Spt6-specific primers. Values were normalized to the quantity of GAPDH mRNA in each sample, and the value for the control sample (scr) was set to 1. Click here for file

Additional file 3 **Figure S3**. Association of Spt6 with the GAPDH promoter is not diminished by ablation of PAAF1. HeLa-LTR-luc cells transfected with the indicated siRNAs were harvested and analyzed by ChIP using anti-Spt6. A sequence within the GAPDH promoter was amplified by Q-PCR. The value for the control (Scr) sample was set to 1. Graph represents mean +/- SD obtained from at least 3 independent experiments. Click here for file

Additional file 4 **Figure S4**. Association of H2B and H3 with the GAPDH promoter is not diminished by ablation of PAAF1. HeLa-LTR-luc cells transfected with the indicated siRNAs were harvested and analyzed by ChIP using the indicated antibodies. A sequence within the GAPDH promoter was amplified by Q-PCR. The value for the control (Scr) sample was set to 1. Graphs represent mean +/- SD obtained from 3 independent experiments. Click here for file

Additional file 5 **Figure S5**. Ablation of PAAF1 induces HIV-1 transcription. U2OS-LTR-MS2 cells were transfected with pMS2-cherry and pTat-Flag where indicated, then fixed and analyzed by immunofluorescence. Sites of MS2-labelled HIV-1 transcription sites are indicated by arrowheads. Click here for file

Additional file 6 **Figure S6**. Identification of cellular genes controlled by Spt6 and/or PAAF1. (A) Heat map showing the differential regulation of cellular genes following loss of PAAF1 or Spt6, as compared to a negative control siRNA. Results from duplicate experiments are shown. (B) Individual genes within the cancer pathway that were identified by transcriptional profiling were analyzed by IB using the indicated antibodies in HeLa cells transfected with control, PAAF1 or Spt6 siRNA. Click here for file

Additional file 7 **Table S1**. Sequences of oligonucleotides pairs used for q-PCR amplification. Click here for file
