# Supplementary figures and images for: Spt6 levels are modulated by PAAF1 and proteasome to regulate the HIV-1 LTR
Source: Retrovirology. 2012 Feb 8;9:13. doi: 10.1186/1742-4690-9-13 (PMC3305501; doi:10.1186/1742-4690-9-13)

**A**

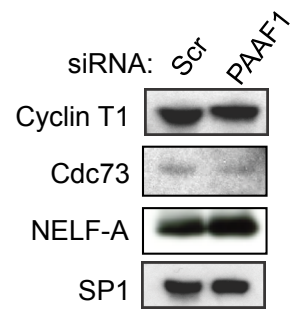

**B**

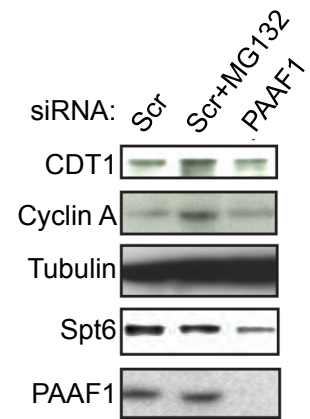

Supplement: Additional file 1 — Figure S1. PAAF1 knock-down does not affect known proteasome substrates or transcription factors other than Spt6. HeLa cells were transfected with siRNA targeting a control (Scr) or PAAF1 (A), followed by treatment with proteasome inhibitor, MG132 (MG), for 8 h before harvesting, where indicated (B). Total cell extract was analyzed by IB using the indicated antibodies. [file 1742-4690-9-13-S1.PDF]

RT-Q-PCR: Spt6/GAPDH

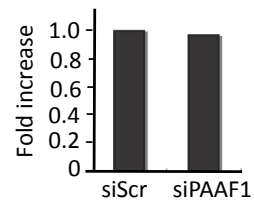

Supplement: Additional file 2 — Figure S2. Spt6 levels are controlled by PAAF1 in a proteasome-dependent manner. Total RNA was isolated from cells in Figure 1A was analyzed by RT-Q-PCR using Spt6-specific primers. Values were normalized to the quantity of GAPDH mRNA in each sample, and the value for the control sample (scr) was set to 1. [file 1742-4690-9-13-S2.PDF]

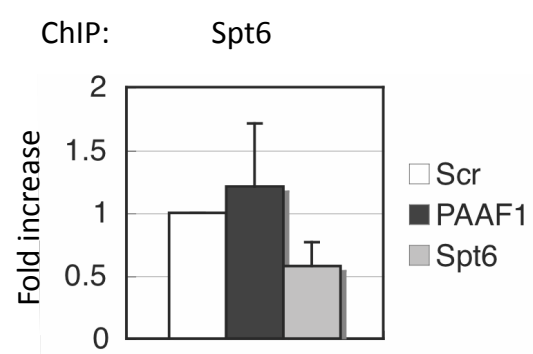

Supplement: Additional file 3 — Figure S3. Association of Spt6 with the GAPDH promoter is not diminished by ablation of PAAF1. HeLa-LTR-luc cells transfected with the indicated siRNAs were harvested and analyzed by ChIP using anti-Spt6. A sequence within the GAPDH promoter was amplified by Q-PCR. The value for the control (Scr) sample was set to 1. Graph represents mean +/- SD obtained from at least 3 independent experiments. [file 1742-4690-9-13-S3.PDF]

GAPDH

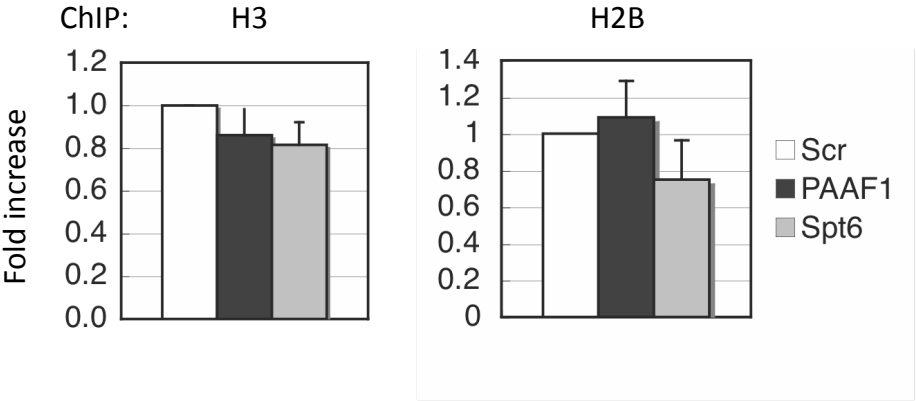

Supplement: Additional file 4 — Figure S4. Association of H2B and H3 with the GAPDH promoter is not diminished by ablation of PAAF1. HeLa-LTR-luc cells transfected with the indicated siRNAs were harvested and analyzed by ChIP using the indicated antibodies. A sequence within the GAPDH promoter was amplified by Q-PCR. The value for the control (Scr) sample was set to 1. Graphs represent mean +/- SD obtained from 3 independent experiments. [file 1742-4690-9-13-S4.PDF]

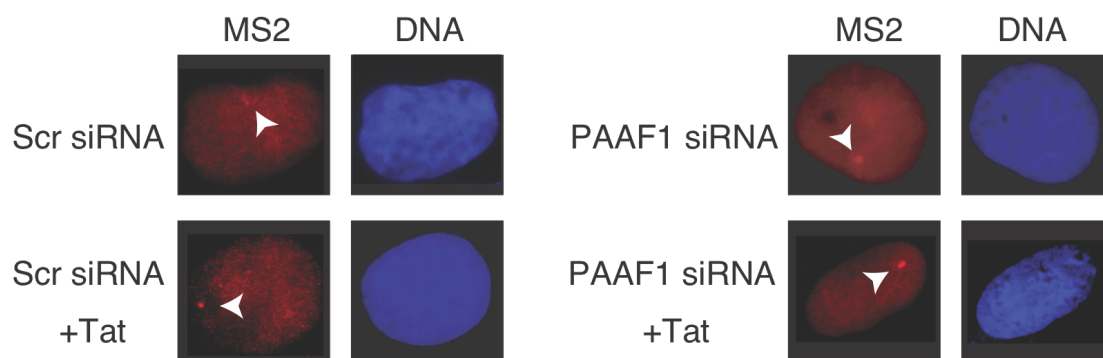

Supplement: Additional file 5 — Figure S5. Ablation of PAAF1 induces HIV-1 transcription. U2OS-LTR-MS2 cells were transfected with pMS2-cherry and pTat-Flag where indicated, then fixed and analyzed by immunofluorescence. Sites of MS2-labelled HIV-1 transcription sites are indicated by arrowheads. [file 1742-4690-9-13-S5.PDF]

**A**

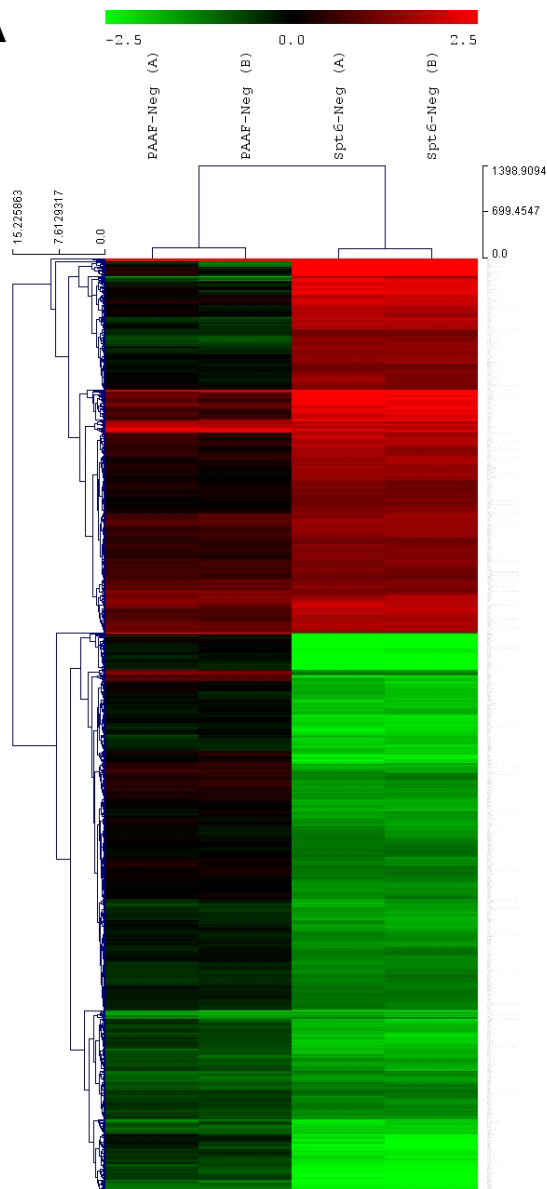

**B**

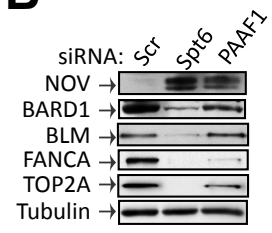

Supplement: Additional file 6 — Figure S6. Identification of cellular genes controlled by Spt6 and/or PAAF1. (A) Heat map showing the differential regulation of cellular genes following loss of PAAF1 or Spt6, as compared to a negative control siRNA. Results from duplicate experiments are shown. (B) Individual genes within the cancer pathway that were identified by transcriptional profiling were analyzed by IB using the indicated antibodies in HeLa cells transfected with control, PAAF1 or Spt6 siRNA. [file 1742-4690-9-13-S6.PDF]
