# Supplementary material for: Spt6 levels are modulated by PAAF1 and proteasome to regulate the HIV-1 LTR
Source: Retrovirology. 2012 Feb 8;9:13. doi: 10.1186/1742-4690-9-13 (PMC3305501; doi:10.1186/1742-4690-9-13)
Supplement: Additional file 7 — Table S1. Sequences of oligonucleotides pairs used for q-PCR amplification. [file 1742-4690-9-13-S7.PDF]

Additional file 7, Table S1. Sequences of oligonucleotides pairs used for q-PCR amplification.

Name	Forward	Reverse
TAR	GGG TCT CTC TGG TTA GA	GGG TTC CCT AGT TAG CC
early	TGG CTA ACT AGG GAA ACC CAC	TTT CAT AGC TTC TGC CAA CC
Luc	AAG AGA TAC GCC CTG GTT CCT G	CGG TAG GCT GCG AAA TGT TCA
Luc mid	ACC CTA TTT TCA TTC TTC GCC	AGC CCA TAT CCT TGT CGT ATC
Luc 3'	TGG CAG GTC TTC CCG ACG AT	GGC GAC GTA ATC CAC GAT CTC T
Spt6	CCA CAC CAA CCA TTT CTC TC	AGG CTC CAC ATA CTC CTT TC
GAPDH	CTG GCG TCT TCA CCA CCA TGG	CAT CAC GCC ACA GTT TCC CGG
SerpinE2	ATC TCC CCC TCT TCC TCT TG	ATT TAC GCC GTA TCT CAT CAC
GDF15	AGA ACT CAG GAC GGT GAA TG	TGG TTA GCA GGT CCT CGT AG
INHBA	ATG TAC CCA ACT CTC AGC C	TCA TCC TCT ATC TCC ACA TAC C
Nov	TTC AGC CAA GCT GCA AAT TCC	CTC CCA GTG AAT CCT CCT CAT C
BRCA1	GAG TAA CAA GCC AAA TGA ACA G	GAG TGC CAT AAT CAG TAC CAG
BARD1	CAA CCA TCT GTT ATC TCC AGT C	GTC ACT ACT TCA TTC CTG CTC
THSD4	CTA CCA GCT ACC TTT GAC CC	TTG CAC AGC TTG TAC TGC C
MET	GTT CAC TGC ATA TTC TCC CC	TTC GTTTCC TTT AGC CTT CTC
E2F1	ATA CCC CAA CTC CCT CTA CC	CTG TCA GTC AGT CTG TCT CC
BLM	TTC ACA CCT TCC CTC AAA TTC	GGC CTT TCA AAA AGA TTC TTC C
FANCA	GCA AAG TCC AGC TCA GTC TC	TCT TAT CTG CCT CTG TCC CC
TOP2A	ACG GGC AAA GAA ACC TAT AAA G	CAG ACA AAG CAG AGA AGA AAA C
PUM1	GTC AAA AGG ACG TGC AAA AG	CCA GGC ATA AGC ATA CAC AG
Prom	CAG CCG CCT AGC ATT TCA TCA C	GGG CAC ACA CTA CTT GAA GCA
Luc coding	AAG AGA TAC GCC CTG GTT CC T	CGG TAG GCT GCG AAA TGT TCA
GAPDH prom	TAC TAG CGG TTT TAC GGG CG	TCG AAC AGG AGG AGC AGA GAG CGA
